# From tracking data to ecologically grounded tactical indicators: a conceptual validation procedure for team invasion sports

**DOI:** 10.3389/fpsyg.2026.1876991

**Published:** 2026-09-03

**Authors:** Zhao Gang, Lin Zixuan, Jiang Yong

**Affiliations:** College of Physical Education, Shenzhen University, Shenzhen, China

**Keywords:** action opportunities, ecological dynamics, eco-physical variables, explainable AI, representative task design, tactical indicators, team invasion sports, tracking data

## Abstract

Tracking systems and AI-assisted models now describe team invasion sports in considerable detail. They estimate distance, speed, pitch control, passing probability, pressure, expected possession value, and many other position-derived variables. The difficulty is not the measurement itself. It is the step that follows, when a spatial or predictive output is written as if it already carried tactical meaning. A passing lane can be geometrically open and still be unusable for the current ball-carrier; a defender can be close without restricting the relevant exit; an action-value model can predict a useful action without showing that the action was locally available in the episode. This article defines E-PART as a five-step conceptual validation procedure for judging whether a candidate tracking-derived variable may legitimately be reported as an ecologically grounded tactical indicator. E-PART stands for Ecological constraints, Positional relationships, Action-opportunity specification, Representative task boundaries, and Tactical emergence. The procedure starts with a neutral position-derived variable and examines the performer-task-environment relation that the variable is asked to represent. It then leads to one of four reporting decisions: report the variable as a bounded tactical indicator, qualify the scope of the claim, downgrade the output to spatial or predictive description, or reject the tactical interpretation. The paper's central claim is narrow: tracking-derived variables become tactical indicators only when the inferential chain from coordinates to action opportunity to tactical consequence is stated, bounded, and open to empirical testing. The contribution is therefore procedural. E-PART makes the move from position-derived measurement to tactical interpretation visible, criticizable, and reportable.

## 1 Introduction

In a routine post-match report, the same player can appear both free and unavailable. A tracking file marks an open passing lane; video review shows the ball-carrier receiving near the touchline with a closed body shape while pressure arrives from the inside shoulder. The measured spatial relation is real. The tactical claim is less secure. The lane exists as a geometric route, but the episode may not support the stronger statement that a forward pass was locally available.

This problem has grown with the sophistication of tracking and modeling. GPS/GNSS, optical tracking, and video-based systems allow analysts to estimate interpersonal distances, synchronization, team center of mass, spatial exploration, pitch control, and pressure-related measures (Memmert et al., 2017; Carrilho et al., 2020; Low et al., 2020; Rico-González et al., 2020; Goes et al., 2021). Machine-learning approaches add formation recognition, pass-probability modeling, action valuation, and pattern detection at a scale that manual coding cannot sustain (Herold et al., 2019; Teixeira et al., 2025). Action-value frameworks such as VAEP and expected possession value are valuable because they estimate the value of many actions rather than only rare events such as shots and goals (Decroos et al., 2019; Fernández et al., 2021). The concern is the unexamined promotion of a measured or predicted relation into tactical language.

Without a screening procedure, two errors are easy to reproduce. The first turns measurability into meaning: a distance, angle, or probability is treated as if the value already explains what a player could do. The second turns predictive success into tactical validity: a model may forecast which actions tend to succeed while leaving open whether the specific action was locally available to this performer, under this pressure, with this body orientation, support structure, and task goal. This matters for researchers who name new indicators, analysts who build dash boards, and coaches who use those dashboards to design training tasks. A weak interpretation can travel from a coordinate value into a training recommendation before the inferential steps have been inspected.

This article proposes E-PART to address that problem. E-PART is a five-step conceptual validation procedure for evaluating whether a candidate tracking-derived variable can legitimately be reported as an ecologically grounded tactical indicator. E-PART stands for Ecological constraints, Positional relationships, Action-opportunity specification, Representative task boundaries, and Tactical emergence. The procedure takes as input a neutral position-derived variable, such as defender distance, passing-lane openness, pitch-control probability, or expected possession value. E-PART then asks five questions: what ecological constraints give the value meaning; what positional relationship is being described; what task-relevant action opportunity is being claimed; within which representative task boundary the claim can travel; and what tactical consequence makes the relation worth reporting. The output is one of four reporting decisions: report the variable as a bounded tactical indicator, qualify the scope, downgrade the output to spatial or predictive description, or reject the tactical claim.

The thesis is narrow but important. Position-derived variables become ecologically grounded tactical indicators only when the performer-task-environment relation, task boundary, and tactical consequence are specified. E-PART makes that inferential chain explicit. The procedure supplies no universal thresholds and leaves empirical validation intact. It tells authors, reviewers, and practitioners what must be stated before a tracking-derived variable can be used as more than a spatial or predictive descriptor.

## 2 Conceptual scope and key terms

This article is a Hypothesis and Theory contribution that defines a procedure for deciding when position-derived variables can carry tactical interpretation in team invasion sports. The contribution is procedural rather than metric-based or empirical-validation based: E-PART sets out what must be specified before a variable is reported as an ecologically grounded tactical indicator. Football provides the main illustrative domain because positional-data research is especially developed there, but the procedure is intended for team invasion sports more broadly. Application to basketball, handball, rugby, hockey, futsal, or lacrosse requires recalibration when timing, density, rules, scoring targets, or player capabilities differ.

A position-derived variable is any coordinate-based or model-estimated description derived from tracking data, such as distance, angle, velocity, acceleration, area, arrival time, control probability, or expected value. A positional relationship is the organized relation among players, opponents, ball, target space, time, and task goal that gives a variable possible tactical relevance. A task-relevant action opportunity is a cautious claim that a measured relation supported, restricted, or altered what a performer or unit could do under the constraints of the episode. A tactical indicator is a variable or compound relation that has passed enough conceptual and empirical checks to support a bounded tactical interpretation.

The terminology is intentionally modest. The central claim concerns task-relevant action opportunities, while perception, intention, anticipation, and decision quality remain separate questions. Tracking data can support claims about timing, orientation, pressure, distance, and likely intervention windows. Coordinates are still indirect evidence of what a player saw, intended, or anticipated. E-PART therefore screens the tactical interpretation attached to a position-derived variable; it does not recover the full perceptual or cognitive process behind the action. This caution is consistent with sport-specific accounts of cognizant action, where skilled performers act prospectively in changing situations but coordinate data remain only partial evidence of those action processes (Araújo et al., 2025).

## 3 Theoretical rationale

### 3.1 From events to continuous goal-directed relations

An event and a tactical opportunity claim answer different questions. A pass, shot, carry, press, or recovery records what happened. A tactical opportunity claim asks what relation existed before or during the event, which performer and task goal the relation concerned, and what consequence could reasonably follow. This distinction fits ecological-dynamics accounts of behavior as an emergent product of performer, environmental, and task constraints (Newell, 1986; Araújo et al., 2006; Davids et al., 2008; Araújo and Davids, 2016). Team-sport accounts of synergies, coordination, and team decision-making have also made relational analysis central to performance interpretation (Duarte et al., 2012; Araújo et al., 2023). The constraints-led approach gives the same problem an applied form: task design and coaching interventions depend on how constraints shape available actions (Renshaw et al., 2019; Chow et al., 2021).

Performance analysis has long recognized that discrete event counts are insufficient for explaining team-sport behavior. The shift from recording discrete actions toward analyzing continuous goal-directed behaviors is explicit in ecological performance analysis (Correia et al., 2013; Travassos et al., 2013). Eco-physical variables develop this point by asking whether metrics preserve goal-directed athlete-environment relations rather than detached spatial summaries (Lopes et al., 2025). E-PART works at a different layer. Eco-physical variables describe the kind of variables ecological performance analysis should seek; E-PART specifies how a candidate tracking-derived variable should be screened before the tactical wording is attached.

### 3.2 Representative task design as a boundary condition

Representative task design gives E-PART its transfer boundary. Brunswik (1956) introduced representative design as a way of preserving the organism-environment relations relevant to the target ecology (Brunswik, 1956). In sport, representative task and learning designs ask whether practice or research tasks retain the informational variables, action possibilities, and constraint relations that support behavior in the performance environment (Dicks et al., 2009; Pinder et al., 2011; Travassos et al., 2012). This matters for tracking-derived indicators because a variable calibrated in one task ecology can lose tactical meaning when moved to another.

A pressure threshold estimated in adult professional football can be technically reliable and still be weak for youth players, a reduced-sided task, or another invasion sport. A passing-lane variable calibrated in an 11v11 match may overstate opportunity in a drill where defenders are constrained, or understate opportunity in a counter-attacking context where body orientation and timing differ. Ecological approaches to practice design show the same transfer problem in applied settings, where tasks must be fitted to the information-action relations of the target performance environment rather than copied by surface resemblance (Woods et al., 2020; Yearby et al., 2024). E-PART therefore treats transfer as a property of the task ecology rather than as a universal property of the metric.

### 3.3 Analytics, prediction, and conceptual validation

Sport analytics has produced powerful tools for describing and valuing behavior. Passing-network analysis captures circulation and connection. Pitch-control and spatial-influence models estimate likely control of space. Action-value and expected-possession frameworks estimate the value of observed or potential actions (Decroos et al., 2019; Fernández et al., 2021). Explainable AI approaches can help analysts inspect which inputs contribute to a model output, and recent reviews show that explainability is becoming a general issue across sport science rather than a football-only concern (Kranzinger et al., 2025). Recent football studies have also connected tracking data with tactical interpretation and defensive success prediction (Forcher et al., 2024; Teixeira et al., 2025).

These approaches are useful, but they answer a different question from E-PART. Prediction asks whether a model forecasts an outcome. E-PART asks whether the relation on which the model or metric depends can support the tactical language attached to the output. A high-value potential pass may still be locally unavailable if pressure, body orientation, receiver timing, or defender arrival makes the action impractical in the episode. Conversely, a low-probability action may reveal a meaningful tactical relation if the model omits a constraint that practitioners recognize. Conceptual validation therefore complements modeling by checking what modeling results may legitimately be said to mean.

### 3.4 Measurement-level contribution of E-PART

Many familiar positional measures occupy an awkward middle ground. Team center of mass, nearest-defender distance, and pitch-control surfaces describe location or influence. Tactical use requires specifying who could act, toward which target, before which opponent intervention, and with what consequence. A passing-route relation, for example, may need ball-arrival time, defender-intervention time, passer orientation, receiver orientation, and the target zone after reception before the tactical claim is warranted.

AI-assisted analysis can preserve the same gap. Pass-probability and action-value models may be useful while staying silent about why an option existed, why the option was ignored, or whether a comparable relation could be practiced in a representative task. Feature attribution can show that distance, angle, or speed influenced a prediction. E-PART checks the next step: whether those features specify a performer-task-environment relation. The procedure locates this missing step and gives it a reportable structure.

The risk is conceptual as well as technical. Ecological dynamics can become a vocabulary rather than a measurement change if relational terms are attached to variables detached from capabilities, task goals, and representative boundaries. E-PART prevents this drift by forcing the claim back to specific relations, evidence levels, and reporting decisions.

### 3.5 Positioning E-PART within conventional validation pathways

E-PART should be read as a conceptual screening procedure before and alongside empirical validation that complements established validity evidence. Technical reliability, construct validity, criterion validity, ecological validity, predictive validity, and practitioner usability remain necessary. E-PART enters earlier and remains available later. The procedure asks whether the proposed tactical interpretation is conceptually specified enough to deserve empirical testing and careful reporting. Table 1 positions E-PART within conventional validation pathways and clarifies how conceptual screening complements technical, construct, criterion, ecological, predictive, and usability evidence.

**Table 1:** Positioning E-PART within conventional validation pathways.

Validation layer	Main question	What conventional evidence establishes	E-PART contribution
Conceptual screening before/alongside empirical validation	Does the proposed variable have a defensible tactical claim?	Usually not addressed directly; often assumed during indicator construction or model interpretation.	Specifies the performer-task-environment relation, opportunity claim, task boundary, and tactical consequence before tactical language is used.
Technical reliability	Can the intended relation be measured with sufficient precision?	Device error, calibration quality, ball-tracking precision, and stability of orientation or velocity estimates.	Clarifies which relation must be reliable; defender distance alone may be irrelevant if pressure angle is the real claim.
Construct validity	Do values change when theoretically relevant constraints change?	Sensitivity to pitch size, starting positions, pressure, support, task rules, or player capabilities.	Identifies which constraints should be manipulated because they are part of the tactical claim rather than generic covariates.
Criterion validity	Do indicator labels agree with expert or practitioner readings?	Agreement with annotated pass availability, pressure restriction, line-break potential, or other expert labels.	Makes expert disagreement interpretable by locating disagreement at the relation, opportunity, boundary, or consequence.
Ecological and transfer validity	Does the indicator hold in the target task ecology and beyond the calibration context?	Evidence that information-action-constraint relations are preserved across match, drill, age, level, or sport contexts.	Requires the transfer boundary to be stated; unsupported generalization leads to qualification or downgrade.
Predictive validity and usability	Does the indicator add forecasting value and support decisions?	Out-of-sample prediction, model comparison, analyst-coach feedback, decision logs, or intervention studies.	Prevents predictive usefulness or dashboard clarity from being mistaken for tactical meaning.

Table 1 locates E-PART in the validation pathway. Table 2 later controls the wording and downgrade of opportunity claims; Table 3 outlines empirical validation routes. Sensor accuracy and predictive performance belong to other validation layers. E-PART decides whether the tactical interpretation attached to a variable is sufficiently specified to be reported, qualified, downgraded, or rejected.

**Table 2:** Action-opportunity evidence ladder and reporting decisions.

Evidence level	Minimum evidence	Permissible wording	Downgrade rule
Level 0: spatial descriptor	Distance, angle, area, or model probability without specified performer-task relation.	Spatially open lane, close defender, control surface, predicted option.	Do not call the relation a tactical opportunity.
Level 1: relational candidate	Focal performer, ball, opponent, teammate, target, timing frame, and direction of play are specified.	Candidate passing route, candidate pressure relation, candidate receiving state.	Report as a candidate relation unless timing and accessibility are also shown.
Level 2: local action opportunity	Relational geometry plus timing evidence, such as ball-arrival time vs. defender-intervention time or time-to-contact.	Locally available pass, locally restricted exit, locally usable receiving option.	Qualify if orientation, pressure direction, or next-action state is unknown.
Level 3: bounded tactical indicator	Timing evidence plus feasibility proxies, including body orientation, pressure direction, receiver orientation, cover, or next-action consequence.	Bounded tactical opportunity under stated phase, population, and task ecology.	Keep the boundary visible; do not generalize beyond the calibrated ecology.
Level 4: transferable indicator	Level 3 evidence replicated across comparable episodes, task manipulations, levels, or sports with explicit recalibration.	Transferable tactical indicator for a defined family of tasks.	Transfer requires recalibration when timing, density, scoring target, rules, or player capabilities change.

**Table 3:** Hierarchical validation pathway for E-PART indicators.

Level	Question	Evidence
Technical reliability	Is the tracking system sufficient for the intended relation?	Device error, calibration records, ball-tracking precision, orientation accuracy, comparison with criterion system.
Variable reliability	Do repeated measurements give comparable relational values?	Test-retest checks, coefficients of variation, sensitivity analysis, data-provider comparison.
Construct validity	Do values shift when relevant constraints are altered?	Changes in pitch size, starting positions, task rules, pressure, support options, or team numbers.
Criterion validity	Do labels agree with expert readings of play?	Expert or practitioner coding, ROC/AUC, kappa, disagreement review, annotated examples.
Ecological validity	Does the task preserve the game relation being claimed?	Comparison of informational, actional, and constraint correspondence with the target environment.
Predictive validity	Does the indicator add explanatory or forecasting value?	Regression, mixed-effects models, machine-learning tests with confound control and out-of-sample evaluation.
Transfer validity	Does the indicator hold beyond the calibration task?	Cross-level, cross-format, or intervention studies followed by match or match-like assessment.
Practitioner usability	Does the output support an analytic or training decision?	Think-aloud interviews, usability tasks, decision logs, analyst-coach feedback.

## 4 The E-part procedure

E-PART consists of five checks applied to a candidate position-derived variable. Use begins with two short statements. First, the variable is named in neutral language, for example defender distance, passing-lane openness, pitch-control probability, or expected possession value. Second, the intended tactical claim is written beside it: pressure trap, locally available forward pass, usable space, progression opportunity, or another game-relevant interpretation. The gap between these two statements is what E-PART screens.

The five checks then narrow the claim. Ecological constraints identify the conditions that give the value meaning. Positional relationships specify what the coordinates actually organize. Action-opportunity specification states what could be done and what evidence supports that claim. Representative task boundaries mark where the interpretation may travel. Tactical emergence connects the relation to a consequence such as a line break, forced backward pass, turnover, entry, shot, recovery, or stabilization. After these checks, the claim is reported, qualified, downgraded, or rejected. Table 4 summarizes the five E-PART checks, the core validity question attached to each check, and the common misreadings the procedure is designed to prevent.

**Table 4:** E-PART checks, validity questions, assessment routes, and common misreadings.

Check	Core validity question	Assessment route	Common misreading
E: ecological constraints	Which performer, task goal, opponent relation, environmental condition, and performance level give the variable meaning?	State performer, task, and environmental constraints; identify phase of play, role, pressure, pitch region, and competition level.	Treating a threshold as universal.
P: positional relationships	How are coordinates organized into action-relevant relations rather than isolated locations?	Specify focal performer, ball, opponents, teammates, target space, timing, and direction of play.	Equating geometry with tactic.
A: action-opportunity specification	What task-relevant action opportunity is being claimed, and what evidence supports the claim?	Name the action, performer capability, timing requirement, and observable support for reachability or use.	Calling open space a tactical opportunity without performer-task evidence.
R: representative task boundary	Where can the interpretation travel, and where should the claim be qualified?	Check informational, actional, and constraint correspondence with the target environment.	Generalizing from a drill, dataset, league, or age group without boundary conditions.
T: tactical emergence	What tactical consequence makes the relation worth reporting?	Connect the indicator to line breaks, forced backward passes, turnovers, entries, shots, recoveries, or stabilization.	Reporting a value without naming the tactical phenomenon that the value helps explain.

### 4.1 Ecological constraints

The first check prevents variables from floating free of the conditions that give them meaning. The analyst identifies the focal performer or unit, the phase of play, the task goal, relevant opponents and teammates, environmental constraints, and the level or population. A two-meter defender distance can indicate severe pressure in one episode and little pressure in another, depending on approach angle, cover, ball-carrier orientation, pitch region, and available support.

Constraints function as the conditions under which a relation may, or may not, support a tactical opportunity. In practice, analysts can use contrastive logic: hold the positional value roughly constant and ask whether a change in player level, body orientation, field size, defensive starting position, support distance, or time pressure changes the tactical reading. If the same distance no longer means pressure once orientation or support changes, the value should not be used as a stand-alone threshold.

### 4.2 Positional relationships

The second check asks how coordinates are organized into relations. Team center of mass, nearest-defender distance, and pitch-control surfaces become tactically interpretable only when linked to the players, ball, target space, time, and task goal that make the relation actionable. The point is not to add complexity for its own sake. The point is to state what the measure is about.

A positional relationship should be treated as candidate evidence rather than as a tactical indicator in itself. Geometric specification is necessary but insufficient. A passing lane may look open while the receiver is facing backward or while a defender closes the passer's dominant foot. A spacing advantage may be visible while the ball-carrier lacks the body orientation to use the space. E-PART keeps this distinction visible by asking what task-relevant action opportunity the relation could support and where its interpretation should stop.

### 4.3 Action-opportunity specification

The third check names the candidate opportunity in modest language: 'a forward pass was locally available,' 'the first defender restricted the inside escape,' or 'the receiving state supported forward progression.' Performer capability and timing are part of this claim. The analyst does not need access to every perceptual or cognitive state of the player, but the wording should show what is inferred and what remains unknown.

In practice, the analyst can treat action-opportunity specification as a four-step inference. First, identify the candidate action, such as a forward pass, line-breaking carry, or pressing recovery. Second, specify the relational geometry that could support the action: distance, angle, relative velocity, and player-ball-opponent configuration. Third, add accessibility evidence, including ball-arrival time relative to defender-intervention time or time-to-contact. Fourth, qualify the claim with execution-feasibility proxies, such as body orientation, pressure direction, dominant-foot access, or receiver orientation. If only the first two steps are available, the output should remain a spatial or relational description. Table 2 provides the action-opportunity evidence ladder and specifies how claim wording should be downgraded when evidence is incomplete.

The ladder is a wording discipline rather than a scoring scale. When evidence is incomplete, the claim should move downward from tactical opportunity to local relation, spatial descriptor, or predictive output.

### 4.4 Representative task boundary

The fourth check states the scope of the claim. A variable can be informative inside one task ecology and weak outside that ecology. Reduced-sided games, conditioned drills, youth competitions, and professional matches differ in speed, space, technique, tactical culture, and available information. E-PART therefore treats representative design as a reporting obligation. Authors should specify which information-action-constraint relations are preserved, which relations are removed, and how the change affects transfer.

A small-sided game may reveal local pressing relations, but full-team pressing claims in 11v11 require evidence that the relevant information-action-constraint relations are preserved. Laboratory isolation may preserve selected visual information while removing live perception-action coupling. Match play preserves the richest ecology and the least experimental control. E-PART uses representative task design to mark the indicator's boundary: which tactical opportunity claims the task can support, which claims exceed it, and what must be recalibrated before transfer to another sport, age group, or task format.

This matrix turns representative design into a reporting rule. Authors should name the correspondence that is preserved and the correspondence that is lost. The lost correspondence determines how far the tactical wording must be qualified. Table 5 summarizes the representative task boundary dimensions and the evidence required to support transfer of E-PART claims across task ecologies.

**Table 5:** Representative task boundary matrix for E-PART claims.

Boundary dimension	Core question	Evidence to report	If weak or absent
Informational correspondence	Does the task preserve the visual, spatial, temporal, and opponent information used in the match claim?	Field of view, ball speed, opponent starting position, support location, time pressure, and relevant target space.	Downgrade to a task-specific relation; do not infer match opportunity from surface geometry alone.
Actional correspondence	Does the task preserve perception-action coupling and the actions available to the performer?	Live opposition, pass/carry/shoot options, body orientation, first-touch demands, and consequences of action.	Qualify as a drill or laboratory indicator; avoid claiming match transfer.
Constraint correspondence	Do rules, pitch dimensions, player numbers, score incentives, fatigue, and tactical phase match the target ecology?	Pitch size, player numbers, rules, phase of play, age/level, tactical culture, and competitive pressure.	Recalibrate thresholds and report the population/task boundary explicitly.
Tactical-consequence correspondence	Does the task preserve the consequence that gives the relation tactical meaning?	Line break, forced backward pass, turnover, entry, shot creation, recovery, stabilization, or loss of progression.	Report description or prediction only if no tactical consequence can be linked.

### 4.5 Tactical emergence

The fifth check prevents pattern detection from becoming its own endpoint. A tactical indicator should connect to a recognizable tactical phenomenon: line break, progression, forced backward pass, pressing trap, recovery, shot creation, defensive instability, or possession stabilization. This check also protects against over claiming. A model output may be statistically useful while remaining tactically under-specified if the relation does not map onto a consequential pattern of play.

A tactical phenomenon should meet a minimal evidential standard before the phenomenon is used for this check. The phenomenon should be nameable in applied performance-analysis language, linked to a subsequent tactical consequence, and open to change through a task constraint identified in earlier E-PART checks. A high centrality value in a passing network, for example, may describe involvement, but the value does not by itself demonstrate that the player created or used a tactical opportunity. Tactical emergence keeps the indicator tied to the game problem that made the variable worth measuring.

### 4.6 Reporting decisions

E-PART produces four reporting decisions. A report decision is appropriate when the candidate relation is technically reliable, relationally specified, task-bounded, and connected to a tactical consequence. A qualify decision is appropriate when the relation is meaningful but only under stated constraints, such as a particular age group, pitch size, team phase, data provider, or tactical culture. A downgrade decision is appropriate when the variable remains useful for spatial description or prediction but does not justify tactical language. A reject decision is appropriate when the candidate relation is too weak, ambiguous, or detached from the task goal to support the proposed interpretation.

**Figure 1:**
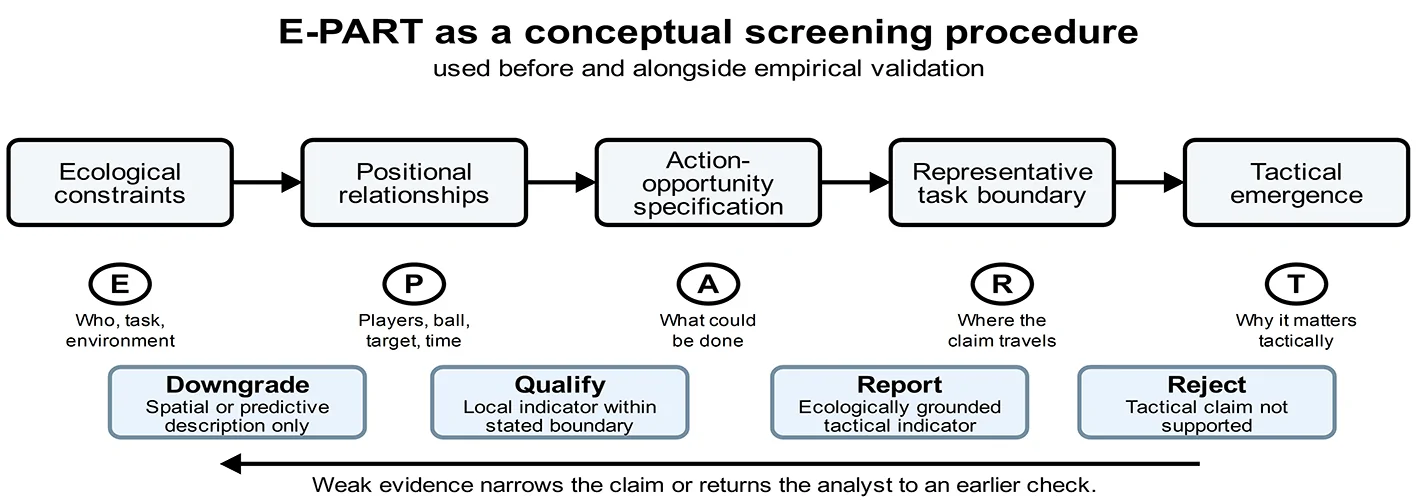
The E-PART conceptual screening procedure. E-PART screens a candidate tracking-derived variable before and alongside empirical validation. The claim is progressively specified, bounded, and connected to tactical emergence. Weak evidence narrows the claim or returns the analyst to an earlier check.

This decision logic matters because the purpose of E-PART is to make claims smaller when the evidence is smaller. A pass-completion probability can remain a valid predictive output while being downgraded as evidence for a local action opportunity. A distance threshold can remain a descriptive pressure proxy while being qualified to a specific tactical context. A failed E-PART check is useful because the failed check shows where interpretation exceeded evidence. The overall screening logic and reporting flow are summarized in Figure 1.

## 5 Worked probes and evidence pathway

### 5.1 From ordinary variables to bounded tactical indicators

Operationalizing E-PART begins with the unit of appraisal. The most useful unit is usually the episode-level candidate relation, such as a ball-carrier-defender-receiver-goal configuration or a pressing unit-ball-carrier-touchline configuration. This unit is less compact than a possession-level label but closer to the tactical claim being made. The analyst then parses the candidate into five fields: focal performer or unit, relevant environmental relation, task goal, action-opportunity claim, and observable tactical consequence. Table 6 illustrates how ordinary position-derived measures can be reframed, qualified, downgraded, or upgraded through E-PART.

**Table 6:** Ordinary measures, E-PART limitations, and bounded tactical indicators.

Candidate measure	Why insufficient alone	E-PART-supported version	Evidence required
Nearest-defender distance	Distance alone does not show whether the defender can restrict the player.	Pressure-opportunity indicator: distance + approach angle + closing speed + cover + ball-carrier orientation.	Level 2 to Level 3: expert pressure coding; angle, speed, cover, and orientation; forced-action outcomes, turnovers, or backward passes.
Open passing lane	A geometric lane may be undeliverable or tactically weak after reception.	Passing-opportunity indicator: route openness + passer orientation + time-to-intercept + receiver state + next-action possibility.	Level 2 to Level 3: pass-availability annotation; time-to-intercept; passer/receiver orientation; progressive reception and line-break outcomes.
Pitch-control surface	Spatial influence is not equivalent to usable opportunity for a specific performer.	Usable-space indicator: control probability + arrival timing + actor capability + task goal + consequence.	Level 1 to Level 3/4: local calibration; actor capability and arrival timing; expert validation; tactical entries, shots, stabilization, or progression outcomes.
Passing-network centrality	Describes involvement or connectivity, not a local opportunity.	Not sufficient unless linked to episode-level performer-task opportunities.	Usually Level 0-1. To reach Level 2/3, link centrality to an episode-level performer-task relation and consequence.
Expected threat/action value	Predicts value of actions or locations but may not show local availability.	Candidate tactical indicator only when linked to performer-specific opportunity and task boundary.	Predictive Level 0/1 unless availability is specified. Level 3 needs expert annotation, task boundary, and tactical consequence evidence.
Ball-arrival/interception timing	Timing helps, but does not alone specify passer, receiver, and defender relations.	Pass-interception indicator: ball-arrival time relative to defender-intervention time + performer capability + receiver next-action state.	Level 2 timing evidence; Level 3 adds capability, receiver next action, expert coding, and defender-starting-position manipulation.

Evidence levels refer to Table 2.

### 5.2 Passing-route opportunity

The touchline example exposes a common analytic misclassification. A midfielder receives near the touchline, and a striker moves between two center-backs. On a pitch map, the route appears open. In the game, the pass is not played. A model may code the lane as open or assign the pass a reasonable completion probability. E-PART asks a narrower question: did the measured relation support a task-relevant passing opportunity for this ball-carrier under the constraints of the episode?

A first-pass E-PART specification would begin with ball-arrival time and defender-intervention time, then add passer body orientation, pressure direction, receiver separation, receiver orientation at arrival, and whether reception would support a forward next action. This specification extends the action-possibility logic of futsal passing-task research, where representative practice depends on available possibilities for action and action fidelity, and aligns with eco-physical variables by treating passing routes as goal-directed performer-environment relations rather than detached geometric paths (Travassos et al., 2012; Lopes et al., 2025). The claim should be downgraded if orientation, timing, or post-reception consequence is unavailable. In that case, the analyst may report a geometric passing lane or predicted completion probability, but not a fully specified tactical opportunity.

**Figure 2:**
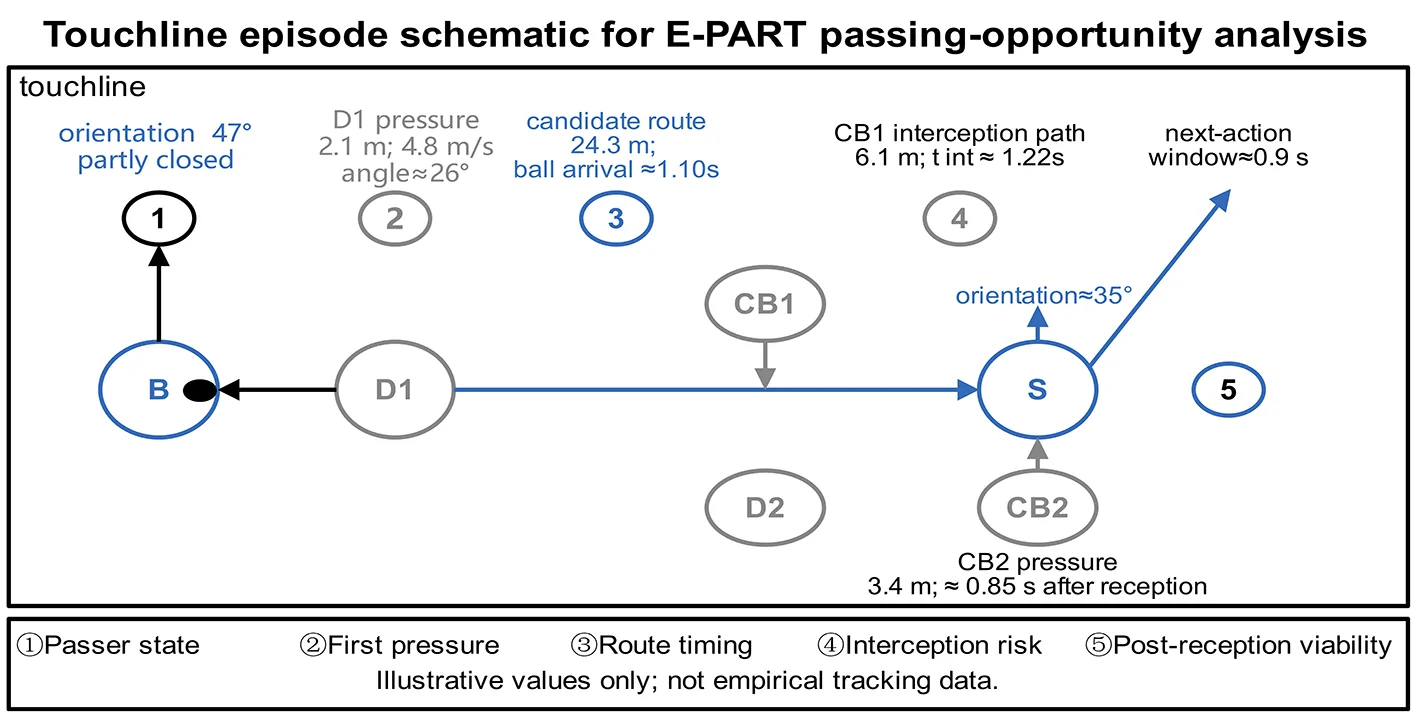
Touchline episode schematic for E-PART passing-opportunity analysis. The figure illustrates how a geometrically open passing lane can be downgraded or qualified when episode-level relations are inspected. The hypothetical values show the types of evidence an analyst would need to consider: passer orientation, first-defender pressure, route timing, cover-defender interception risk, receiver orientation, and post-reception pressure. The values are illustrative rather than empirical tracking data. The schematic shows the inferential structure of E-PART and does not validate the indicator empirically.

Figure 2 gives a schematic version of the same episode. The numerical labels are illustrative and hypothetical rather than empirical tracking data. The purpose is to show the inferential structure that a later annotated tracking study would need to test. If the analyst only has route geometry, the episode should remain a spatial passing-lane description. If timing, pressure, and receiver state are also specified, the claim can be upgraded to a bounded passing-opportunity interpretation.

The evidence pathway is layered. Technical reliability concerns whether ball path, defender closure, and orientation are estimated precisely enough. Construct validity asks whether the indicator shifts when pressure angle, receiver orientation, defensive starting position, or pitch width changes. Criterion validity compares the classification with expert judgments of pass availability at the episode level. Tactical-emergence evidence asks whether stronger values correspond to line breaks, progressive receptions, or reception states that allow the receiver to face forward.

E-PART is not simply a call to add more variables. Extra variables matter only when they reduce a particular uncertainty in the action-opportunity claim. Passer orientation speaks to deliverability; receiver orientation speaks to the next action; defender-intervention time speaks to whether the route survives pressure. A variable that improves prediction but says little about performer, task, timing, or consequence has not strengthened the tactical claim.

### 5.3 Pressing-trap opportunity

A second worked probe concerns defensive pressure. Nearest-defender distance remains attractive because the value is simple, but the value often hides the relation coaches care about. A defender may be close and still fail to restrict action if the approach angle opens the inside pass, cover is late, or the ball-carrier has already shaped for escape. Conversely, a slightly greater distance may still function as pressure if the defender closes the intended exit with support behind.

An E-PART pressing-trap indicator begins from the restriction relation. The analyst asks whether the first defender closed the intended exit, whether a second defender covered the next pass, whether the touchline or backward body orientation reduced options, whether time-to-contact was shorter than time-to-release, and whether the opponent was forced into a backward pass, uncontrolled clearance, turnover, or loss of forward progression. This indicator is less compact than a pressure radius, but the indicator makes visible the configuration that may have restricted the opponent's action field.

E-PART slows the dashboard workflow, but that cost is part of its value. A team may be close to the ball while repeatedly leaving the inside channel open. A reduced-sided drill may appear to improve pressing because the boundary closes escape routes that 11v11 match play leaves available. A youth team may need different thresholds because first touch, passing range, and recovery speed alter what counts as actionable pressure.

For reporting, the pressing-trap example should be phrased with its evidence level visible. A weak claim might state only that the defending team achieved close proximity around the ball. A stronger but still bounded claim might state that the first defender restricted the inside exit while cover behind reduced forward options. A still stronger claim would connect that restriction to forced backward passes, recoveries, or loss of forward progression across comparable episodes. The wording of the indicator should change as the evidence changes.

### 5.4 Evidence hierarchy and reporting standard

E-PART uses an evidence hierarchy rather than a weighted score. A score would imply agreed weights for reliability, ecological validity, transfer, and practitioner usability, which the field currently lacks. A validation program should define the system of analysis, test whether the task preserves the target information-action-constraint structure, manipulate key constraints, and inspect whether the indicator shifts in theoretically expected directions. Expert annotation and practitioner review should separate preferred from non-preferred patterns rather than allowing numerical regularity to stand alone.

Table 3 sketches empirical work that can follow an E-PART claim. The levels are logical evidence points rather than a mandatory temporal sequence.

Reporting should preserve the trade-offs. Alongside the indicator, authors should state task format, player numbers, pitch dimensions, competition level, coordinate processing, candidate thresholds, orientation estimation, annotation rules, and the tactical consequence being inferred. Terms such as viable pass, defensive cover, or pressing trap require explicit annotation rather than intuition. Shared protocols and short annotated examples would improve comparison across sports and levels while still requiring local calibration.

## 6 Discussion

The main implication of E-PART is conservative. Some variables may have to be demoted rather than renamed. A value can describe, predict, or communicate a pattern and still fall short of tactical interpretation. E-PART gives that weakening a place in reporting by narrowing the claim to a performer, task format, level, or tactical phase.

This point does not weaken advanced analytics. Passing networks, expected possession value, action-value models, pitch-control surfaces, and XAI systems remain useful. E-PART asks what their outputs can be said to mean tactically. A high-value potential pass may still be locally unavailable; a pitch-control advantage may still fail to specify who could use the space, when, and toward what consequence. In other cases, a modest model output can become tactically informative once the relation and task boundary are made explicit.

### 6.1 E-PART and explainable AI as complementary tools

The relationship with explainable AI is best treated as a division of labor. XAI can show which inputs, regions, or moments shaped a model output. E-PART asks a different question: whether those highlighted features form a performer-task-environment relation strong enough to carry a tactical name. Defender distance, angle, or speed may help a model predict an outcome. The tactical issue is whether those values describe restriction, access, support, or consequence in the episode. Used together, XAI and E-PART can produce outputs that are both model-readable and tactically bounded: the predictive features are visible, the task boundary is stated, and the tactical consequence is named.

Distribution shift is also a task-boundary problem. A model trained on one league, age group, tactical culture, or sport may lose force because the action landscape changes. A possession-dominant football model may read counter-attacks poorly; adult professional thresholds may misclassify youth play. Model transfer should therefore be reported as a property of the task ecology, not as a universal property of the metric.

The connection with eco-physical variables is useful here. Lopes et al. (2025) argue that performance analysis should preserve intentional, goal-directed athlete-environment relations. (Lopes et al., 2025)E-PART takes that concern to the review-procedure level by asking whether a candidate relation preserves enough performer, task, environmental, and tactical structure to be treated as an ecologically grounded tactical indicator. Recent critical discussion of ecological dynamics in applied practice points in the same direction: conceptual parsimony and empirical accountability both matter (Collins et al., 2025).

### 6.2 Expertise, calibration, and transfer

E-PART also clarifies the place of expertise. Data production and tactical interpretation are often separated. The procedure asks analysts to state the relation and evidential status, and asks practitioners to judge whether the task ecology matches the performance problem. Cognitive accounts of team coordination remain relevant because tactical behavior extends beyond spatial relations alone (Eccles and Tenenbaum, 2004; Ashford et al., 2021). Recent work on how top-level players view decision-making research also supports keeping practitioner interpretation visible rather than allowing model outputs to stand alone (Magnaguagno and Beck, 2025).

This changes the analyst-coach conversation. Alongside the value, analysts report the relation that made the value meaningful, the task boundary under which the value was calibrated, and the decision that the value is meant to support. Coaches can then disagree with the boundary, not merely with the number. That disagreement can be useful because it moves discussion from whether a dashboard is right or wrong to whether the assumptions behind the indicator match the performance problem.

The main limitation is the status of E-PART as a conceptual procedure rather than an empirically calibrated scoring tool. E-PART provides no universal cut-offs for football, basketball, handball, rugby, hockey, or other invasion sports. Perception, intention, fatigue, and neuromuscular state remain beyond coordinate data alone. Orientation data and contextual annotations can narrow the gap, but those data cannot close it. Future work should test whether E-PART-guided indicators correspond with expert annotation, shift under purposeful task manipulations, and improve feedback, training design, or decision-making in match-like settings.

Because the worked probes use football, transfer should be read as recalibration rather than threshold transfer. Basketball, handball, rugby, hockey, lacrosse, and futsal differ in timing windows, density, legal contact, possession rules, scoring targets, and the meaning of space. E-PART supplies questions that make these differences visible rather than identical thresholds across sports.

Future work should regroup the five checks around calibration, transfer, and practitioner use. Calibration studies can compare opportunity indicators with expert annotations. Transfer studies can manipulate pitch size, player number, starting position, pressure, or rules and test whether indicators move in expected directions. Applied studies can ask whether E-PART-guided feedback changes behavior in match or match-like settings. Evidence will probably accumulate unevenly across sports. That unevenness is itself informative.

## 7 Conclusion

The central problem in applied tactical analysis is the unmarked step from position-derived variables to tactical meaning. E-PART renders that step explicit. The procedure asks analysts to specify ecological constraints, positional relationships, action-opportunity claims, representative task boundaries, and tactical emergence before treating a variable as an ecologically grounded tactical indicator.

The touchline episode captures the issue. A pitch map shows an open lane, yet the pass is not taken. A geometric model confirms that the lane existed. E-PART asks whether the midfielder could deliver under pressure, whether the striker could receive and act forward, whether the ball would arrive before defensive recovery, and whether these conditions hold in the target task ecology. If the answers remain uncertain, the lane should be reported as a candidate spatial relation or predicted option, not as a fully specified tactical opportunity.

E-PART is useful only if researchers allow some indicators to fail and practitioners treat those failures as diagnostic. Failed indicators reveal where a metric loses ecological footing: at the performer, the relation, the task boundary, or the tactical consequence. A validation procedure from which no indicator is ever downgraded provides no meaningful standard. The value of E-PART lies in making tactical interpretation more precise, more modest, and more accountable.

## Data Availability

The datasets presented in this study can be found in online repositories. The names of the repository/repositories and accession number(s) can be found in the article/supplementary material.
